# Knowledge, Attitudes, and Behavior Related to COVID-19 Testing: A Rapid Scoping Review

**DOI:** 10.3390/diagnostics11091685

**Published:** 2021-09-15

**Authors:** Imogen Bevan, Mats Stage Baxter, Helen R. Stagg, Alice Street

**Affiliations:** 1 School of Social and Political Science, University of Edinburgh, Edinburgh EH8 9LD, UK; imogen.bevan@ed.ac.uk; 2 Usher Institute, University of Edinburgh, Edinburgh EH8 9AG, UK; M.S.Baxter@sms.ed.ac.uk (M.S.B.); helen.stagg@ed.ac.uk (H.R.S.)

**Keywords:** COVID-19, social science, social solidarity, isolation, testing

## Abstract

Testing programs for COVID-19 depend on the voluntary actions of members of the public for their success. Understanding people’s knowledge, attitudes, and behavior related to COVID-19 testing is, therefore, key to the design of effective testing programs worldwide. This paper reports on the findings of a rapid scoping review to map the extent, characteristics, and scope of social science research on COVID-19 testing and identifies key themes from the literature. Main findings include the discoveries that people are largely accepting of testing technologies and guidelines and that a sense of social solidarity is a key motivator of testing uptake. The main barriers to accessing and undertaking testing include uncertainty about eligibility and how to access tests, difficulty interpreting symptoms, logistical issues including transport to and from test sites and the discomfort of sample extraction, and concerns about the consequences of a positive result. The review found that existing research was limited in depth and scope. More research employing longitudinal and qualitative methods based in under-resourced settings and examining intersections between testing and experiences of social, political, and economic vulnerability is needed. Last, the findings of this review suggest that testing should be understood as a social process that is inseparable from processes of contact tracing and isolation and is embedded in people’s everyday routines, livelihoods and relationships.

## 1. Introduction

Diagnostic testing has been critical to the global COVID-19 pandemic response. In the early months of the pandemic, and in the absence of effective therapeutics or a vaccine, rapidly developed diagnostic tests were among the few countermeasures available to governments to control the spread of the virus, reduce transmission, and prevent deaths [1,2]. As governments sought to exit national lockdowns, diagnostic tests became important for enabling the reopening of workplaces, schools, and/or retail and hospitality venues and thus for helping to restart the economy [3]. More recently, with the vaccine rollout in many (but not all) countries gaining momentum, tests—whether deployed at borders or in educational settings, workplaces, hospitality and entertainment venues, or community hot spots—have become a valuable instrument for keeping the virus at bay [4,5].

The expectations that COVID-19 testing programs impose on members of the public (in terms of the frequency of testing and the expectation to self-isolate while awaiting results or following a positive result) are unprecedented for medical testing [6]. Testing also often entails significant costs—e.g., in terms of the inconvenience and discomfort associated with accessing and undergoing testing or the personal and economic sacrifices required by self-isolation. Yet, except for the use of tests for triage and care decisions in the hospital setting, most people targeted by testing programs see no personal medical benefits from testing and, in some cases, also have no symptoms of COVID-19 infection. While the benefits of testing are primarily for public health, it is individuals who largely bear their costs. The continued willingness of members of the public to undergo COVID-19 testing is therefore of paramount importance; indeed, it is potentially as significant, in terms of the success of disease control efforts, as are the accuracy and reliability of the tests themselves.

In addition to carrying the social, psychological, and economic costs of undergoing testing, members of the public have had to familiarize themselves with a complex, highly technical, and constantly changing landscape of testing types and formats. The rollout of testing has varied greatly between country settings, with the design of testing programs, the speed of their establishment, and the levels of availability of different test types all influenced by the specific course of the epidemic in each location; by local supply chains; by public resources; and by the extent of existing public and/or private testing infrastructure. Different testing methods have also been deployed for a variety of use cases in each country setting [7,8,9]. In the UK, for example, polymerase chain reaction (PCR) tests, which detect genetic material from the virus, were initially only deployed to test seriously ill patients in hospitals [10] but were later made available for mass symptomatic testing in the community. More recently still, they have been used to identify asymptomatic cases in viral hotspots. Lateral flow antigen tests, by contrast, have most often been deployed to identify asymptomatic cases in workplaces and educational settings [11], while rapid antibody tests have been deployed primarily for population-level surveillance [12].

None of the available tests is perfect [13,14]. Some entail long waiting times for results, and some involve invasive sampling techniques. No diagnostic test is ever able to correctly identify all true positive cases or negative cases. Even bearing this in mind, however, the tests currently in use vary widely in terms of their accuracy [4]. Each test use case is also associated with different criteria for eligibility, different systems for accessing testing, and different guidelines for acting on test results. Where and when community testing is not available or is under resource pressure, for example, people have been asked to self-apply strict eligibility criteria to safeguard vital supplies and protect hospitals. Meanwhile, in contexts of the widespread availability of testing, people have been asked to err on the side of caution and seek tests based on general and vague lists of symptoms. Such lists rapidly become outdated as new variants associated with different disease courses appear. The potential for misunderstanding, uncertainty, and doubt in relation to this rapidly changing and diverse testing landscape is vast.

Given the burden this places on individuals undergoing testing, understanding people’s views of the testing landscape; their motivations to seek testing; their trust in tests, providers of tests, and the authorities who set public health guidelines; and their willingness to shoulder some personal costs in the interests of public health, is key to the design of effective testing programs worldwide [15]. Moreover, as the pandemic continues and vaccine programs help weaken links between infection, hospitalization, and death, the question of how to maintain public motivation to test and follow guidelines for self-isolation and protective behaviors is critical to ensure the continued efficacy of testing programs into the future [16].

Written 19 months on from the development of the first tests [17] to detect the SARS-COV-2 virus, this rapid scoping review reflects on what we have learned so far about the knowledge, attitudes, and behavior of members of the public in relation to COVID-19 testing. Drawing on scoping review methods from health research and the social sciences, it provides a preliminary overview of the size and scope of current research and a narrative analysis of results [18,19]. The study has three goals. First, we seek to provide an overview of the extent and type of research undertaken on knowledge, attitudes, and behaviors related to COVID-19 testing to date. This includes a summary of the main research questions posed by these studies, the most common methods used, the settings in which studies are located, the point in the pandemic when data was collected, and their target populations. Second, we identify some of the key themes that have emerged from research on knowledge, attitudes, and behavior relating to COVID-19, with a particular focus on motivations and barriers to seeking, accessing, and undertaking testing and on the impacts of testing on behavior and wellbeing. Last, we identify the limitations of current research in this area and existing knowledge gaps. We also outline the areas of social science research on COVID-19 diagnostics that we believe should be prioritized for investment in the future. This rapid scoping review is not intended to be a comprehensive systematic review and evaluation of all available evidence on this topic; instead, our aim is to identify common themes and omissions across a rapidly emerging and interdisciplinary field to help researchers contextualize their research findings and inform priorities for future research.

## 2. Materials and Methods

### Literature Search

The databases Web of Science, Medline, and Scopus, as well as the World Health Organization’s (WHO’s) global research database, were searched for research on people’s knowledge, attitudes, and behavior related to COVID-19 testing for the period January 2020 to 26 March 2021. Preprint articles were included in the search to ensure that in a rapidly changing testing landscape, studies focused on the most recent testing developments were represented. Where a preprint was subsequently published, we cited the published reference, including instances when this was published outside our original search date parameters.

Example terms used for the bibliographic search in each database included ‘covid’, ‘test’, ‘diagnosis’, ‘screening’, ‘attitudes’, ‘understandings’, and ‘barriers’ (Supplementary Materials, Table S1). Given the limited timeframe of this study and to minimize irrelevant results, we subsequently limited our search criteria to include only papers containing ‘covid’ and ‘test’ in their titles. Results were imported into Covidence review software, and two independent reviewers (AS and IB) removed duplicates and screened the titles and abstracts of studies against inclusion criteria (Supplementary Materials, Table S2). The full texts of selected papers were independently reviewed by both researchers. Given our focus on covering the scope of current research, articles were not assessed for quality. Selected papers that were deemed highly relevant were subsequently entered into Google Scholar for forward citation searches, and the results were screened for eligibility by both reviewers. The bibliographies of these papers were mined for further literature between 26 March and 31 March 2021, and the results were screened in the same way as above.

Selected references and metadata (publication date, author name, title, and abstract) for included articles were imported into Microsoft Excel. Additional data ‘charting’ [19,20] was undertaken for country setting, study methods, target population, study population, dates of data collection, testing type, and context of testing (community, institutional, symptomatic/asymptomatic). We also recorded the limitations of each study as reported by their authors. Where studies included many components, including the piloting of an intervention, we only recorded those limitations related to research on knowledge, attitudes, and behaviors related to COVID-19 testing. Qualitative analysis entailed identifying common research questions posed in relation to COVID-19 testing followed by thematic coding of study findings under each of these question headings.

## 3. Results

**Figure 1:**
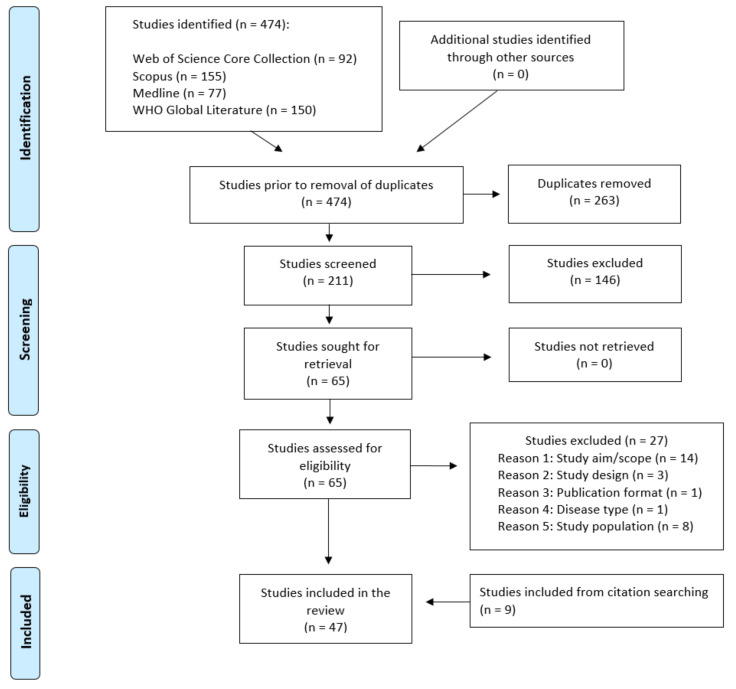
Flow diagram of included studies and reasons for exclusion.

In total, 474 articles and preprints were identified by the databases (Figure 1). After removing duplicates, two independent reviewers (AS and IB) screened the abstracts of 211 papers. Full-text screening was undertaken on 65 articles and preprints, following which a further 27 studies were excluded. Nine additional articles were identified by in-text and forward citation checks. We included 47 papers in the final review.

### 3.1. Overview of Studies

**Figure 2:**
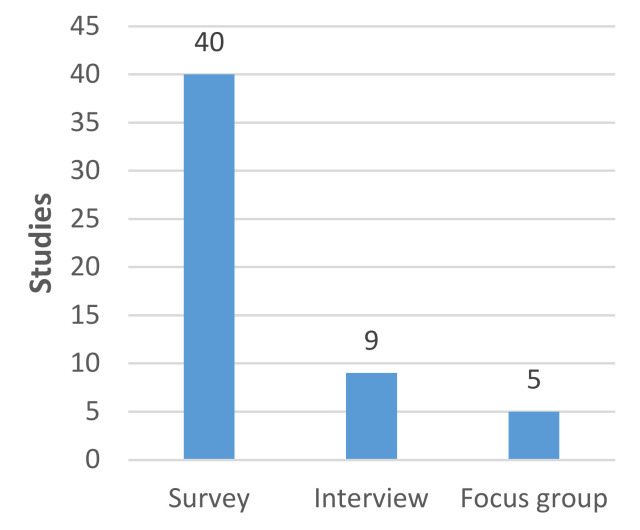
Study methods.

Most of the papers included in the review (n = 47) employed quantitative survey-based methods (Figure 2). Of these, single-event cross-sectional surveys were the most common data collection method. Smaller numbers of case-control surveys (either randomized or cohort comparisons), time-series of cross-sectional surveys, longitudinal surveys, and survey-based discrete choice experiments were represented. Only nine studies employed interview methods. Five studies used focus groups (Figure 2). Due to restrictions on face-to-face contact linked to the pandemic, most of the research was carried out online via online survey software or video and teleconferencing software.

**Figure 3:**
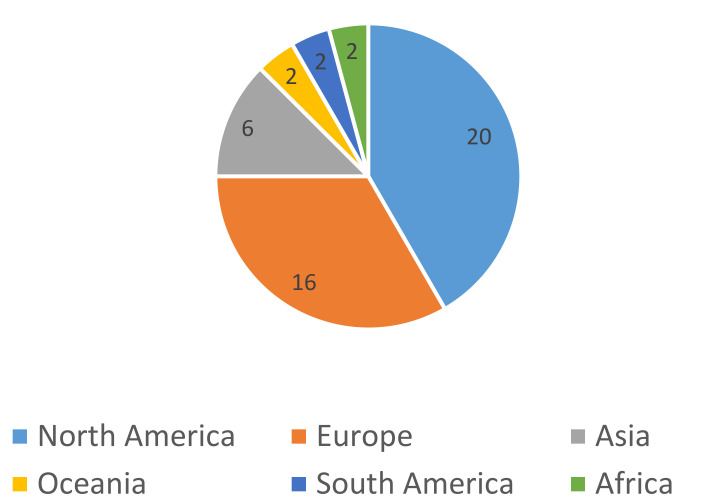
Geographic regions of data collection.

As highlighted in Figure 3, most of the studies were based either in North America (20, of which 19 were based in the US) or Europe (16, of which 13 were based in the UK). Smaller numbers of studies were based in Asia (six), Oceania (including Australia) (two), South America (two), and Africa (two).

Many studies (n = 26) targeted the general public or a geographically defined sub-national population in the country where the research was based. Such studies commonly used social media and internet-based convenience sampling methods to recruit participants. Eight studies [21,22,23,24,25,26,27] targeted participants of pilot testing programs (e.g., a telehealth system for testing [21] or the introduction of mass asymptomatic testing on a university campus [23]). Other target populations included patients or users of a specific health service or clinic [21,28,29] or health-related online app [30,31]; age-based populations [32]; health workers [33,34,35,36,37]; students and/or staff based at universities [23,24,25,26,27,38]; employment groups, such as police officers or industry workers [39,40]; and groups deemed especially vulnerable to COVID-19, such as homeless populations [41], refugee populations [42], or pregnant women attending clinical settings [34,35,43].

**Figure 4:**
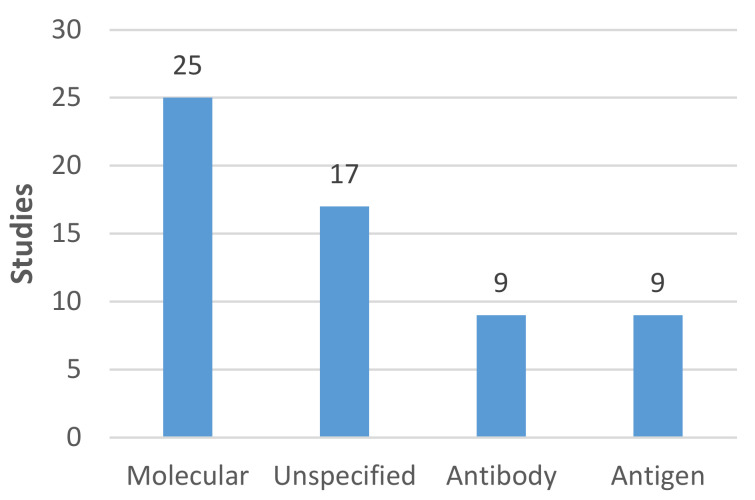
Test type.

Molecular tests were the type of COVID-19 tests most often included in a study (n = 25, all of which except one used PCR tests) (Figure 4). Antibody and antigen tests featured in far fewer studies (n = 9 in both cases). Seventeen studies did not specify the test type. Most studies focused on community-based testing (n = 34); only 14 focused on testing in institutions, of which eight were based in non-healthcare settings (e.g., schools, universities, and workplaces).

**Figure 5:**
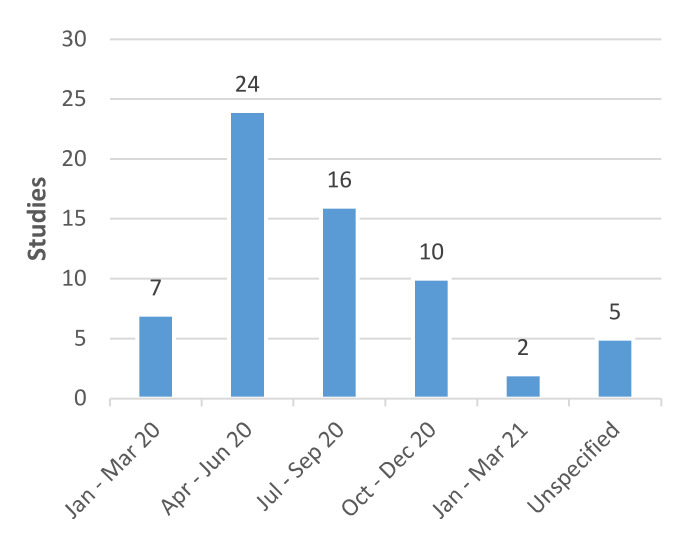
Data collection periods covered by included studies, by quarter.

The earliest month that data collection started was March 2020, and the latest date that data collection ended was January 2021 (Figure 5). The pandemic, and the testing programs that developed in response, unfolded over different timeframes in different locations. Nonetheless, most countries represented in the review were in the midst of their first or second COVID-19 wave during the second and third quarters of 2020, the most common periods of data collection. Missing data on testing format meant that it was not possible to correlate the testing format studied with the stage of the pandemic in each country setting. Nevertheless, the majority of high-income countries represented in the review had established community-based PCR testing for symptomatic cases during this period but had not yet instituted widespread rapid lateral flow antigen testing. Lateral flow testing is less widely represented in the evidence base and is more likely to feature in articles and preprints based on more recently collected data [22,38]. The characteristics of all studies included in the review are summarized in Table 1 [21,22,23,24,25,26,27,28,29,30,31,32,33,34,35,36,37,38,39,40,41,42,43,44,45,46,47,48,49,50,51,52,53,54,55,56,57,58,59,60,61,62,63,64,65,66,67].

### 3.2. Scope of Research

The research questions posed by the included studies fell into two broad areas. First, several studies sought to understand the facilitating factors driving, and the barriers preventing, the acceptability and uptake of testing. Second, several studies sought to understand the impact or effect of testing on subsequent behaviors, attitudes, and experiences.

Studies that explored facilitating factors included research on participants’ knowledge and awareness of testing types and how they work [36,44] and research on people’s identification and interpretation of COVID-19 symptoms, including how this affects test-seeking behavior [30,45,46,47]. Multiple studies in this research area gathered data on people’s self-reported motivations for undergoing or avoiding testing [22,23,24,26,27,29,30,32,35,37,38,40,41,42,45,46,47,48,49,50,51,52,53]. Some of these studies sought to gather data on people’s trust in the government and/or medical systems in relation to COVID-19 testing [24,41,54,55] or piloted ways to improve people’s levels of trust in testing [42]. While the majority of studies focused on self-reported willingness to test, a certain number also sought to measure and explain associations between demographic characteristics and the actual uptake of testing (e.g., according to gender, age, ethnic, geographic, or socioeconomic variables) [22,23,30,37,42,43,45,47,51,56,57,58].

Studies that focused on testing effects and post-testing behaviors included those concerned with the impact of testing—or the perceived availability of testing—on people’s wellbeing and anxiety levels [23,24,27,33,34,35,38,39,40,59]; they also included those examining adherence to guidelines following test results, whether in terms of compliance with self-isolation guidelines in the case of a positive result or of maintaining protective behaviors in the case of a negative result [22,23,28,31,33,38,39,41,45,51,52,57,60,61]. All of the studies under this theme were based on self-reporting; no studies in the review undertook observational research on the impact of testing on compliance with guidelines.

## 4. Thematic Findings

**Figure 6:**
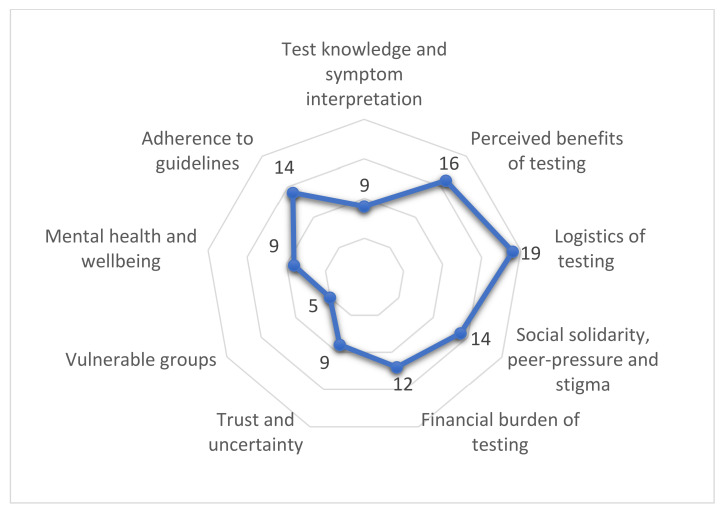
Themes.

Under each of the categories for study scope (facilitators/barriers and testing effects), we identified a series of sub-themes related to findings from the included studies. The spread of themes is visualized in Figure 6 below. Specific findings related to each theme are summarized in Table 2 and Table 3.

### 4.1. Acceptability, Uptake, and Barriers to Testing 

#### 4.1.1. Test Knowledge and Symptom Interpretation

Study results show that, since the start of the pandemic, members of the public in multiple countries have rapidly come up to speed with a complex typology of testing technologies and formats for the SARS-COV-2 virus and have, in many cases, gained a general understanding of the main symptoms and clinical presentation of the COVID-19 disease [36,44,53]. Nonetheless, the most important finding under this theme was an association between people’s interpretation and (mis)recognition of symptoms and their test-seeking behavior, in many cases relating to their failure to seek testing when symptomatic [30,42,45,46,47,49]. For example, a longitudinal survey of members of the UK public in January 2021 (n = 53,880) found that just 22.2% of participants who had self-reported experiencing symptoms in the previous seven days also reported requesting a test [45]. Also in the UK, a large-scale longitudinal survey of COVID-19 symptom tracking-app users (n = 3193) found that a quarter of participants who logged symptoms and would have qualified for a test in December 2020 did not undergo testing [30].

Studies that explored this theme found a knowledge gap around symptom identification and eligibility. The research team for the UK longitudinal survey study, for example, found that only 51.5% of participants knew the key symptoms of COVID-19 communicated by the UK government (high temperature, cough, and, from May 2020, loss of taste/smell) [45]. This highlights the challenge of applying binary criteria (e.g., symptoms are present or absent) to individuals’ subjective experience of illness. Reasons for not booking a test identified by the above study included ‘symptoms had improved (16.9%)’ and ‘symptoms were only mild (16.3%)’ [45] (p. 6). The longitudinal study of UK app users, moreover, found that ‘Testing was lower with one vs. more symptoms (73.0% vs. 85.0%), [and with] short vs. long symptom duration (72.6% versus 87.8%)’ [30] (p. 3).

Another common reason for not seeking a test while symptomatic was that participants did not think the symptoms were caused by COVID-19 [42,45,46,47]. In a survey of the general population in Saudi Arabia (n = 6378) in October and November 2020 and a study of the general public in the Philippines in August 2020 (n = 147), researchers found that people were more likely to seek testing for symptoms they perceived to be specific to COVID-19 (such as loss of taste/smell or shortness of breath) but less so for other symptoms [46,49]. Participants dismissed other symptoms as indicative of flu or a common cold [46] or reported that they did not think they were experiencing COVID-19 symptoms because they had not had recent contact with anyone who had the virus [45].

Overall, these findings show that people’s knowledge and experience of their own symptoms are re-evaluated in the context of other illnesses, the character and evolution of symptoms, and their experiences of exposure. This suggests that for members of the public, the process of applying testing eligibility criteria to their subjective experiences of illness involves substantial interpretive work on their part.

#### 4.1.2. Perceived Benefits of Testing

We identified three main areas of perceived benefit from testing. First, in many cases, research participants mentioned wider, community-level benefits from undergoing testing, including helping to reduce transmission [22,28,38,40,52] and protecting vulnerable groups [22,28]. An interesting finding from a US-based study using a hypothetical field experiment with 1000 members of the public in May 2020 was that the people most willing to take a test were those most likely to spread COVID-19 (due to high levels of social contact in their daily lives). The authors of that study concluded that people have an altruistic approach to testing for COVID-19 [52].

In many cases, research participants also identified personal benefits from testing, including lower risk of being personally responsible for spreading the virus to others [38,40,52]; knowing their disease status [22,47]; preventing the spread of disease to family members [23,40,61], their unborn fetus [35], or workplace colleagues [39]; and being able to return to work [33]. The personal benefits of testing were especially prominent for people who saw themselves as personally more at risk from COVID-19, including because of age, health status, or ethnicity [38,53,55].

Another perceived benefit of testing was the generation of data that could contribute to scientific research and help the government manage the pandemic. This was especially the case for the perceived benefits of antibody testing [61]. Two UK studies of institution-based testing found that participants felt pride in participating in national efforts to manage the pandemic through taking part in pilot testing programs [23,24].

Overall, these findings suggest that people widely understand that testing primarily benefits people other than the person undergoing testing and that a concern for others and a desire for social solidarity are key motivators for getting tested. Even where study participants identified personal benefits from testing, these were often related to a desire to act responsibly towards others.

#### 4.1.3. Logistics of Testing

The logistics of accessing and undergoing testing were identified as important enablers or barriers to testing. Factors identified here included knowledge of testing systems, technical issues in accessing testing, access to testing sites, processes of sample extraction, and test-to-result turnaround times.

In four studies, members of the public reported confusion and uncertainty regarding how to access or where to go for testing, suggesting that public awareness of testing systems is an important factor in testing uptake [30,47,48,50]. In particular, participants reported not knowing where to go to get tested [30,47,50]. One study of app users and survey respondents in the UK and the US found that not knowing where to go for testing was associated with older age groups, lower levels of formal education, and lower access to smartphones, suggesting possible links between awareness of testing systems and social, political, and economic marginalization [30]. 

Bureaucratic or technical barriers to accessing testing were mentioned in three studies [22,24,25]; for example, problems with the internet and/or technology affected 6% of a group of participants undergoing daily testing (n = 319) among the UK public in December 2020 [22].

Access to testing sites could also be a problem, lack of transport being one obvious barrier [30,50]. Ensuring practical access to testing was found to be especially important for vulnerable groups. A US interview study of homeless populations conducted from July to October 2020 (n = 94) found that ‘mobile teams were convenient because they reached people who did not want to abandon their belongings or leave their neighbors to participate’ [41] (p. 6). Patients with disabilities raised concerns in open text responses in one study, including the need for testing centers to cater to their disabilities or being homebound [48]. A US study survey in September and October 2020 (n = 3058) found that black, male, or young members of the public were among those more likely to want or need a test, but that black and/or male individuals were ultimately less able than white and/or female individuals to access one [47] (p. 1).

In terms of public preferences around testing sites and locations, pilot studies and service evaluation studies that asked about the convenience of their testing sites reported high levels of satisfaction across a range of testing venues and locations. These included home testing, drive-thru, walk-through, community testing, workplace, and campus testing [21,25,41,62,63,64]. A minor concern for participants in some studies was the fear of contracting the virus at the testing center [24,48]. This affected 5% of participants in a survey of the general public in Australia (n = 1369) conducted between April and July 2020 [48].

Several studies found that the convenience and comfort of the sample extraction method affected willingness to test: participants found multiple options (cheek swab or spit, pharyngeal swab, nasopharyngeal swab, finger prick, dried blood spot card) acceptable, but overall preferred less invasive sampling methods [25,62,63,64,65]. Experience of physical discomfort while undergoing testing, especially nasopharyngeal swabbing, was widely reported [22,35,37,43,62], with one study suggesting that the deep nasopharyngeal swabbing method could act as a possible deterrent [62]. This reason was given by 10 out of the 16 patients who declined a test in a survey of patients in a US delivery unit conducted in May and June 2020 (n = 270) [43]. By contrast, a UK study of students in October 2020 found no consensus on preferences for saliva or throat swabs, concluding that physical discomfort did not deter students from participating in a pilot testing program [23].

Negative perceptions of test-to-result turnaround times were a focus of three studies [47,50,62], with one US survey (n = 1221) in July and August 2020 finding that ‘time delays created a perception that testing was futile’ [50] (p. 5). Conversely, a survey across 16 Latin American countries (n = 5504) in March and April 2020 found that people valued longer waiting times (at least in theory), possibly associating them with more accurate types of tests [66], while some participants in a UK study ‘interpreted a longer wait as being suggestive of a positive result’ [25] (p. 5).

Overall our findings suggest that the logistical organization of testing from start to finish, including transportation to test sites, booking systems, sample extraction method, and turnaround times, plays an important role in test-seeking behavior and especially affects access to testing by vulnerable groups.

#### 4.1.4. Social Solidarity, Peer-Pressure, and Stigma

Social pressure (e.g., relating to the expected reactions of employers, friends, and family to an individual’s decision to seek testing or test result) was a sub-focus of 14 studies in this review [23,24,26,27,32,33,38,40,45,46,48,54,67]. Six studies focused on negative pressure from employers and peers [38,40,45,46] or stigma [54,67] as barriers to testing. Negative pressure in institutions included dismissive attitudes towards COVID-19 transmission and pressures from management and colleagues not to seek out testing. In a study of UK police officers (n = 18) in May and June 2020, for example, seeking testing was associated with ‘skiving’ [40] (p. 11). In Malawi, a study of the general population in May 2020 (n = 4641) found that 81% of survey respondents expected to be treated badly if they tested positive for COVID-19 [54]. Negative peer pressure was also a recurrent theme in studies involving student participants, since students are keenly aware that a positive test result will require peers (and often members of a shared household) to self-isolate [27,38].

Consistent with the above findings, a range of studies found that endorsements from and/or solidarity with peers, families, and supportive institutional cultures encourage testing [23,24,26,32,33]. This was especially true in institutional settings (e.g., schools and GP surgeries), where participants’ decisions to engage in a pilot project were ‘influenced by a pull on their sense of community’ [24] (p. 9) and the feeling that they were ‘in it together’ [23] (p. 22) There is some ambiguity about the relative influence of negative and positive pressure in these studies, however, since participants were also reported to fear the stigma associated with a positive test result [24].

The challenge of navigating competing social pressures and obligations in relation to testing was also investigated in an interview-based study of 15 healthcare workers awaiting a test result in Denmark conducted in March and April 2020 [33]. Here, testing created dilemmas in terms of feeling responsible for shifting one’s workload onto colleagues, and yet, complying with guidelines by staying home while waiting for the test result was experienced as a form of solidarity and contributed to participants’ sense of professional identity [33]. The concern that testing could, conversely, undermine social solidarity was raised in one UK study carried out in April and May 2020 (n = 60), with participants fearing that antibody testing and potential antibody passports could create a divided society [61].

These findings suggest that individuals face countervailing negative and positive social pressures and that decisions to test or to self-isolate following a positive result are therefore often experienced as ethical dilemmas. Overall, social endorsement and/or a sense of social solidarity with others are found to be strong motivators for testing.

#### 4.1.5. Financial Burden of Testing

Studies that examined whether or not financial considerations posed a barrier to testing showed conflicting results. Most research in this area took place in the US and UK, where symptomatic testing was formally available free of charge irrespective of medical insurance status. Two quantitative studies, one in the UK (n = 778, conducted from April to June 2020) and one in the US (n = 897, date unspecified), concluded that socioeconomic variables (measured through proxies such as education level, professional expertise, and impact on one’s employment due to COVID-19) were not significantly related to people’s willingness to test [52,55]. Two other US quantitative surveys reported the exact reverse. The first (n = 6378, April 2020) demonstrated that socioeconomic status influenced people’s perceptions of whether they could access testing, with students, unpaid workers, and those on low incomes feeling especially unable to do so [56]. The second (using longitudinal methods) found that 17.9% of individuals who wanted a test but did not take one (n = 1956) had felt unable to afford the cost of the test [30] (p. 9). US-based studies found that whether patients had medical insurance was a key factor affecting access [43] or perceived access [56] to testing. Other approaches included asking people how much they would be willing to pay for a test kit. One such study, conducted in March and April 2020, estimated that in Latin America, ‘The median person would be willing to pay at least 4.2% of the average monthly income for a COVID-19 test (or $45)’ [66] (p. 4), even with a three-day turnaround time for results.

One factor to bear in mind with research on the cost of testing is that financial costs often relate to logistical considerations, such as transportation or scheduling time off work, in addition to the cost of the test kit itself. For example, a survey in the US found that nursing home staff, who must take a test once a month according to new guidelines, do not always have access to workplace testing and therefore may face significant barriers in terms of time and financial costs [37].

A few studies referred to the financial costs associated with self-isolation as a potential concern or disincentive affecting people’s decisions to undergo testing [24,45]. A focus group study, part of a pilot project on saliva testing carried out between June and October 2020 in the UK, found that people were concerned about the possible consequences of testing positive: ‘If they had to isolate they would lose income, their employer would be unsympathetic’, and that ‘a history of infection with the virus might affect their ability to get a mortgage and life insurance’ [24] (p. 11). Importantly, one UK longitudinal study also found that ‘financial hardship, index of multiple deprivation, lower socioeconomic status, and having a dependent child in the household’ [45] (p. 4) was associated with a lower likelihood of requesting a test.

The cost of testing to society was also examined in a study in early April and May 2020 of the UK public’s understanding of COVID-19 antibody testing. Focus group participants (n = 60) expressed doubts about the public cost of testing out of concern that these funds could be better used elsewhere—in vaccine research, for example [61].

Research in this area considered a wide range of costs associated with testing, including logistical considerations and the impact of self-isolation. Some studies show that people are willing to test regardless of socioeconomic status. Others suggest that socioeconomic status can have an important impact on the perceived affordability of testing and that perceptions of affordability influence people’s willingness to test, even where those concerns are not reflected by actual costs.

#### 4.1.6. Trust

Concerns about trust were represented in numerous studies and spanned the whole testing process, from the providers of testing and the process of sample collection to the accuracy of tests and interpretation of test results.

Three studies identified participants’ trust (or the lack of it) in testing providers, governments, and medical systems as a potential barrier to testing [24,42,54]. For example, one case-control study carried out in May 2020 (n = 4641) compared trust in public clinics versus trust in the World Health Organization (WHO) as providers of testing in Malawi. Those authors found that ‘Malawians expect higher community uptake of testing when the agency offering the tests is the WHO rather than a public health clinic’ [54] (p. 4), an effect they attribute to concerns around confidentiality and procedural integrity. In the UK, a pilot feasibility study of saliva testing in schools, a university, and an NHS trust run from June to October 2020 (n = 223) found that participants’ main reason for not taking part was that they did not trust the government with their data and ‘were anxious about the possibility of losing control of their data when the program passed them to NHS Test and Trace in the event of a positive test’ [24] (p. 10).

Six studies explored participants’ confidence in the accuracy and reliability of COVID-19 tests themselves [36,41,54,61,64,65]. In particular, the perceived frequency of false positives affected people’s desires to be tested [38,41,61]. For example, false positives and concerns about the safety of tests were identified as a barrier in an interview study of US homeless populations (n = 94) conducted between July and October 2020 [41]. A focus group study of perceptions of antibody testing in the UK in April and May 2020 (n = 60) similarly found that a small number of people would feel hesitant about taking a test with 98% accuracy because of concerns about false positives and false sense of security this would give [61]. Conversely, a study of lateral flow test uptake on a university campus in the UK in December 2020 and January 2021 (n = 232) found that participants ‘mostly accepted that tests would not be 100% accurate’ [38] (p. 10). Participants, in this case, viewed testing as one measure of one’s likelihood to be infected among others, including their health status and history of social contact. Other studies measured people’s confidence in their ability to correctly collect a specimen (dried blood spot, saliva, throat swab) [65] or to correctly interpret an antibody test result [64], both finding people to be confident in their abilities.

Overall, research in this area shows that trust is a multivalent factor that encompasses people’s relationships with health providers, institutions, technology, and their own abilities. The breakdown of trust in any of these relationships can affect people’s willingness to test.

#### 4.1.7. Vulnerable Groups

Some studies identified barriers to testing that were highly specific to the social, political, economic, or health status of particular sub-population groups. For example, an interview study of homeless populations in the US conducted between July and October 2020 (n = 94) found that fear of loss of shelter could be a deterrent to testing [41]. Another survey, carried out in a refugee camp in Bangladesh (n = 222) from July to October 2020, found high levels of testing refusal among the Rohingya and members of a host community (109 of 222 patients). It concluded that ‘challenges to testing are likely to persist unless considerable efforts are made to address rational fears around testing relating largely to the complex history of the Rohingya population, and to more proximal and immediate fears of lock-down or disclosure of test results’ [42] (p. 2).

Reflecting the unique vulnerabilities associated with maternal health, a survey in July 2020 of 297 asymptomatic pregnant women in Japan found that only half of them had taken a prenatal PCR test [35]—48.8% were ‘concerned regarding the disadvantages of receiving positive prenatal PCR results, such as the possibility of changing delivery facility, giving birth by cesarean section, not meeting neonate or not breastfeeding, and being isolated during hospitalization’ [35] (p. 4). In a four-stage study of pregnant women in a delivery unit in a US setting (n = 270), meanwhile, authors found that women who were either Black or had Medicaid insurance were significantly more likely to refuse testing initially [43].

Only a small number of studies in our review focused on sub-populations associated with vulnerable characteristics, but those that did found important associations between specific vulnerabilities and willingness to test.

### 4.2. Impact of Testing on Attitudes, Behaviors, and Wellbeing

#### 4.2.1. Mental Health and Wellbeing

The relationship between testing and mental health was a common theme across nine studies [23,24,27,34,35,38,39,40,59] and encompassed impacts on mental health associated with the availability of testing, undertaking testing, and post-testing experiences.

Studies largely reported the perceived availability of testing—regardless of whether a participant undertook a test—to be beneficial for mental health and wellbeing [23,24,35,38,39,40,59]. For example, a study of the state of mental health of the general public in Malaysia in May 2020 (n = 669) found that perceived test unavailability predicted rates of anxiety and depression [59]. Access to testing in the workplace or place of study was found to be especially reassuring [23,38,39]. Elsewhere, an interview study of UK police officers (n = 18) in May and June 2020 found that participants expected testing to help ‘combat some of the fear and anxiety generated from working with the virus in close quarters on a day to day basis’ [40] (p. 8) at a time when testing was not widely available in the community. University-based studies reported similar results [23,38]. For example, in a study of students in the UK in October 2020, participants reported lower anxiety and perceptions of being safer and better supported by the university [23,38]. Just one study, a survey of pregnant women in a US delivery unit in May 2020 (n = 560), contradicted these findings by reporting very low levels of reassurance post-testing [34].

Overall, approaches to mental health predominantly focused on questions of reassurance from testing and were especially well represented in studies of institutional testing. Key findings were that access to testing programs provides reassurance at a time of widespread uncertainty and anxiety, and that reassurance is also linked to the knowledge that other people are participating in testing programs in addition to the personal benefits of undergoing testing.

#### 4.2.2. Adherence to Guidelines

Fourteen studies examined the links between testing and self-isolation and/or changes to protective behaviors following a positive or negative test result [22,23,27,28,31,33,38,39,45,51,52,57,60,61].

Several studies reported high rates of intention to self-isolate among their participants [38,60] and of self-reported adherence to guidelines to self-isolate in the home following the positive result of the participant [57] or another member of the household [51]. In contrast to these findings, studies that recorded people’s self-reported behaviors on a day-to-day basis [22,31,45] and studies that compared people’s intentions and words to their practices and actions [45] were more likely to report low levels of self-adherence to isolation and testing behaviors. In some cases, particular socio-demographic characteristics were found to be associated with non-adherence. A UK-based longitudinal survey undertaken in 2020 (n = 53,880) found levels of adherence to guidelines on testing, self-isolating, and sharing contact information to be lowest among men and younger people [45]. A simulation study of 1194 adults in the US general population (data collection date unspecified) found that participants’ health status and political leanings affected their intentions to self-isolate [60].

Several studies noted specific challenges around self-isolation in the context of communal living. In an interview study in Denmark of members of the public (n=15) waiting for PCR test results, participants were often reluctant to follow government guidance on isolating from other household members prior to receiving a test result [28]. In a focus group study of students in the UK in October 2020, some participants reported unclear guidelines about using communal areas in student dorms after a positive test [27]. Finally, one UK survey of the general public (n = 96) from January to March 2020 found that 97% of respondents (n = 89) reported having self-isolated at home following a positive test; however, 41% (n = 38) had not been able to avoid contact with other members of the household [57].

Contact with people from outside the household during self-isolation was also reported. A comparative study of people isolating vs. undergoing daily testing after being contact traced in the UK in December 2020 and January 2021 found that 19% of people self-isolating reported leaving the house [22]. Seven participants with positive test results (out of 54; 13%) reported close contact with people outside the household [22]. Elsewhere, a large-scale longitudinal survey in the US conducted in April and May 2020 (n = 4759) found that people mostly reported staying at home following a positive test result, with the exception of 7% who left home to work [31].

In terms of the impact of negative test results on protective behaviors, two studies—one based in the US, one in the UK—found that people were more likely to engage in risky behaviors following a negative PCR test [22,60]. Two further UK studies—one on lateral flow testing among staff and students at a university in December 2020 and January 2021 (n = 232), the other of asymptomatic PCR testing on a university campus (n = 140) in October 2020—both reported participants’ increased confidence in undertaking permitted activities within existing guidelines following a negative result [23,38].

With regards to antibody testing, researchers found no significant change in behavior following receipt of test results [39,61].

Overall, research on post-testing behaviors shows that widespread self-reporting of intentions to isolate following a positive test is contradicted by findings suggesting lower levels of actual compliance. Studies also report the practical challenges people face in complying with self-isolation guidelines. There is some indication that negative test results lead to fewer protective behaviors, but from the limited evidence available, this does not appear to extend to breaking public health guidelines in the populations studied.

## 5. Reported Limitations of Included Studies

Many of the survey-based studies reported low response rates and potential enrollment bias, pertaining especially to the over-representation of groups who were more worried about COVID-19 or more observant of guidelines. Very few studies that measured the uptake of testing captured the experiences of participants who had refused a test offered to them [35,42] or for which they were eligible [45].

COVID-19 restrictions meant that many of the studies recruited participants online, often through convenience sampling, and therefore excluded groups without access to the internet or digital skills (e.g., elderly participants, children). Several studies reported their failure to include significant numbers of participants from marginalized and at-risk groups such as ethnic minorities, low-income groups, and elderly populations. Findings from studies that focused on sub-groups such as health workers, students, and university employees, reported limitations of not being able to generalize their findings to the wider population of that country. Several studies reported a higher number of female than male participants, which may reduce the generalizability of their findings to men. Many studies reported only very low numbers of participants who had tested positive for SARS-COV-2. This meant that generalizable findings were in most cases limited to the knowledge, attitudes, and behaviors of participants who had either not been tested or who had tested negative for the virus.

Another common limitation of both survey- and interview-based studies was the potential for social desirability bias in self-reporting (either of past behavior or future intentions) and their inability to document actual behaviors. Recall bias was less of a problem because most studies were carried out in rapid response mode and asked questions about very recent behaviors. Many survey-based studies reported the limitations of a multiple-choice format, which prevents further probing of respondents’ experiences and thus inhibits the researchers’ access to the meaning(s) and/or personal significance of participants’ statements and choices. Several qualitative studies reported the limitations of internet- or telephone-based interviewing and the difficulty of establishing intimacy and rapport with participants.

Some surveys also reported the limitations of cross-sectional study design for inferring causation. No included studies measured for the impact of external events and developments in the pandemic—e.g., an increase in infection rates or the greater availability of testing—on people’s knowledge, attitudes, and behaviors. Furthermore, several studies reported that participant attitudes may have changed since the research was completed in response to new epidemiological developments and/or changes to government guidelines and policies.

## 6. Discussion

### 6.1. Thematic Analysis and Recommendations

Thematic analysis of the findings of studies in this review reveals several key discussion points in terms of the social dynamics of COVID-19 testing, with important implications for testing program design and government policy in this area.

**Symptom interpretation is a complex social process:** COVID-19 testing programs are unprecedented in terms of the work that members of the public are expected to do to determine whether testing is necessary and whether eligibility criteria for testing are met, in most cases without any assistance or guidance from medical experts. The review found the application of formal symptom criteria to subjective illness experiences to be a complex process requiring substantial interpretative work on the part of members of the public. Moreover, the non-specific nature of COVID-19 symptoms made this work especially difficult. Individuals resolve these difficulties by incorporating their knowledge of symptom severity and duration and their history of social contact with others. Rather than viewing the finding that many people with formal symptoms do not seek testing as their ‘failure’ to test, we argue that it is important to recognize the complexity of the medical work that people are expected to undertake. We suggest that testing guidelines acknowledge the ambiguities entailed in identifying symptoms and provide additional support and advice to guide people’s decisions.

**People recognize that testing primarily benefits others:** COVID-19 testing differs from many of the other kinds of diagnostic tests that people are familiar with because it is associated with few clinical benefits for those undergoing testing. The review found that people have a good understanding of the societal benefits of testing and that they are often motivated to test to protect others. In other words, people are broadly willing to accept the personal costs and burdens of testing in order to contribute to a communal pandemic response. This finding supports the view expressed by high-profile social psychologists in the UK, that social science research should examine why the great majority of people continue to adhere to COVID-19 regulations despite a lack of structural support, as opposed to focusing on breaches of guidelines [16]. However, further research is required to understand whether such social motivations continue to be prominent in later stages of the pandemic and whether the perceived benefits of testing change in relationship to the course of the pandemic in particular countries and/or the alteration of government policies on vaccines, isolation, and testing.

**People face logistical barriers at multiple stages of the testing process:** The evidence base shows that while people found COVID-19 testing services to be acceptable in most contexts, there are logistical challenges associated with every stage of the testing process—from booking a test or self-application of the test, to transport to a testing site, to receiving the results. Logistical difficulties present particular challenges to people with social, health, economic, or political vulnerabilities. We suggest that a whole-systems approach be taken to design testing programs to ensure that the full spectrum of potential logistical barriers to testing is known and managed at the outset. Findings around the discomfort of sampling methods suggest that easy sampling methods, such as saliva testing, should be prioritized at the development stage of new testing products.

**People are concerned about the impact of their decision to test on others:** While COVID-19 testing has public health benefits, it can also entail sacrifices by the person undertaking testing or by their social contacts. The papers included in this review identified both negative social pressures that are a barrier to testing and positive pressures that encourage it. We suggest that in-depth qualitative research into the everyday ethics of testing decisions and the social dilemmas that people face in deciding to test or to act on a positive result is needed to inform the design of future testing programs and public communications around testing.

**The cost of testing is about more than the cost of the test:** While one study in our review focused on the cost of test kits, other studies revealed that the affordability of testing includes costs associated with transportation, time off work, and self-isolation. The full spectrum of costs incurred by different people, especially those in lower socio-economic income groups, needs to be incorporated into the design of testing programs and government policies on compensation. It should also be acknowledged in public communications around testing. Interventions such as enhanced benefits to support self-isolation could improve the uptake of testing in vulnerable groups and encourage post-test adherence to guidelines.

**Trust is important at every stage of the testing process:** The studies that we reviewed in this area suggested that trust is multifaceted and pertains to multiple relationships across the testing process. The breakdown of trust in any of these relationships—whether trust in the providers of testing, the test itself, or confidence in one’s capacity to self-test—can present a barrier to testing. We argue that further research is required in this area and that the design of testing programs needs to take the full range of social relationships involved in testing into account.

**Social, economic, and political vulnerabilities can affect access to testing and its acceptability:** We found surprisingly few studies that examined social, health, economic, or political vulnerabilities in relation to testing, which we discuss further below. The few such studies we did find, showed that vulnerability in relation to political status, shelter, and maternal health each generated unique challenges for accessing and/or undertaking testing and/or acting on test results. These span concerns of disruption to living situations, changes in medical care arrangements, and mistrust in government linked to long histories of oppression and discrimination. The design of testing programs should always take difficulties of access associated with vulnerability and socio-economic inequality into account.

**Testing programs have the potential to contribute to improving mental health and wellbeing during a pandemic:** Research around testing and mental health found that the availability of testing has the potential to substantially reassure people in the midst of a pandemic. This appears to be especially important in institutional settings such as universities or workplaces, where people find it difficult to avoid social contact with others. Since studies in this area remain fairly narrowly focused on questions of reassurance, and those reviewed here were conducted in the early stages of the pandemic, there is an opportunity for further research to explore relationships between testing and mental health more broadly and at different stages of the pandemic. For example, how might relationships between mental health and testing evolve in the context of widespread vaccination campaigns and changing perceptions of the risks associated with the virus and self-isolation?

Willingness to adhere to guidelines on isolation and protective behaviors is high, but people often face social and practical barriers in practice: Several of the papers reviewed found high levels of self-reported willingness to adhere to guidelines on protective behaviors and self-isolation. However, studies focusing on how individuals actually behaved and the challenges they faced revealed that people often found it impractical or undesirable to follow those guidelines in practice. In particular, self-isolating in communal households was found to be difficult, and many people leave their homes while self-isolating. Rather than taking a punitive approach to the flexing of rules on testing and self-isolation, we suggest that guidelines on testing need to be better informed by what is practical and possible for people in different country settings and socio-economic contexts. Country-specific research in this area would help governments identify policies and actions to help support behaviors that protect public health.

Cutting across these thematic findings, we make three overarching arguments related to the social conceptualization of testing alongside recommendations for future research priorities.

First, our findings show the importance of understanding testing as a social process that is informed by people’s social values and relationships at every stage—from interpreting symptoms, to accessing and undertaking testing, to receiving and acting on the results. We, therefore, argue for a whole-systems approach to testing research and program design that recognizes how vulnerability, trust, affordability, and logistics play out across the different stages of the testing process and not only at the moment a test sample is extracted. In particular, we see a need for more studies to examine relationships between tests and interventions such as self-isolation beyond self-reported intentions. If we consider the full extent of the testing process, including the decision to test/not test, then further research is also needed around the refusal of testing; most studies in this review targeted people who had, or who had not yet, been tested. Understanding testing hesitancy is crucial for designing effective testing programs and is likely to become more important as societies begin to open up, case numbers increase, and growing numbers of people are vaccinated.

Related to the importance of understanding testing as a social process, we argue that research methods (longitudinal/qualitative) are needed that capture people’s experiences across time or provide experience-near accounts of people’s understanding of testing in the context of their social relationships, livelihoods, and daily routines. The current evidence base is dominated by cross-sectional surveys, which limits the ability of researchers to infer causation (e.g., between testing and actual post-testing behaviors), probe the meaning of individual responses, or contextualize people’s responses in relation to their social and economic circumstances. In many cases, the results of survey-based studies are too vague to be useful (many, for example, use people being ‘concerned’ about COVID-19 as a variable, with no further breakdown of specific concerns or their underlying causes [66]). Likewise, most surveys offered a predefined selection of responses and did not investigate in more depth why people sought tests. This points to the need for further qualitative research designed to gain a better understanding of people’s experiences, motivations, and rationales for testing attitudes and behaviors. For example, very few studies considered the ethical dilemmas that people face when balancing guidance telling them to get tested against personal needs or the needs of family/household members, or how they might resolve such dilemmas.

Second, while we identified vulnerability as a standalone theme in the literature, the matter of vulnerability is also relevant for all the thematic areas identified above. In particular, vulnerabilities linked to people’s social, economic, health, and political status have the potential to shape the context in which people experience and interpret symptoms, the benefits they see in testing, ethical dilemmas related to testing, the affordability of testing, trust relationships, their mental health, and their adherence to guidelines. Vulnerability has been an important theme in debates since the start of the COVID-19 pandemic. In some usages, vulnerability has referred to the higher clinical risks associated with certain biological profiles, including pre-existing medical conditions, age, and genetics (in some cases linked to race and/or ethnicity). In the UK, for example, an official list was quickly drawn up, classifying those deemed most clinically vulnerable to the effects of COVID-19, as identified through a risk assessment process, and special guidance on ‘shielding’ for each category issued [68]. In other usages, vulnerability refers to socio-economic factors that affect people’s risk of exposure to the virus or of adverse outcomes—a kind of vulnerability often understood to unfold along ethnic and racial lines, or in line with particular occupations, experiences of inequalities, deprivation, everyday living conditions, and access to transport [69]. However, social scientists have argued that this distinction between biological and social causes of vulnerability is irrelevant, with biological vulnerabilities also often a consequence of the inequitable distribution of resources and privileges within (and between) societies across time [70]. In particular, some researchers insist on the importance of resisting notions of ‘intrinsic Black vulnerability’ [71] (p. 1), and instead argue that ‘The conditions of labor and daily life produce ill-health, and social exclusion and discriminatory attitudes discourage access and undermine health care’ [72] (p. 673) beyond the COVID-19 pandemic. There is an urgent need for social science research that engages vulnerable communities at the same time as critically evaluating the changing meanings and implications of vulnerability in infectious diseases responses [70,73]. Rather than viewing vulnerable groups as compliant or non-compliant in terms of testing behaviors, further research is needed to understand the specific barriers people in these situations may face—taking their concerns seriously rather than merely seeking to establish behavior change. While we recommend that access to testing be improved for vulnerable groups, we are mindful that focusing on questions about access risks glossing over questions of ‘structural vulnerability’ [74] related to ill-health and risk of exposure.

Moreover, we find that the existing evidence base on vulnerability and testing is narrow in scope and depth. The vast majority of research in this review was limited to North America and Europe, and even within that, there is a striking lack of diversity, with study participants overwhelmingly white, middle-aged, and middle-class. Disability status was recorded in just one instance [55], while the kinds of barriers people with disabilities may have faced were not a focus in any of the research we reviewed. The majority of survey-based studies targeted national populations. However, in selecting participants from groups who have access to the internet and a level of digital literacy, their recruitment and data collection methods selected more privileged groups and systematically excluded certain other populations (e.g., the elderly, homeless, or economically disadvantaged) who were also most at risk from COVID-19) [73]. Despite the early emphasis on care homes and nursing homes as crucial sites of COVID-19 transmission, we found very little research carried out in these settings. Other institutional settings associated with populations with socio-economic vulnerabilities, such as prisons, detention centers, and rehabilitation centers, were also excluded.

Third, the review found that knowledge, attitudes, and behaviors related to testing were highly specific to country settings, the course of the pandemic in different locations, and the particularities of governmental responses in different locations. We found that no research currently exists comparing the rollout of testing in different country settings or plotting that rollout against the course of the pandemic in individual countries. It would be beneficial for social science research on attitudes to testing in individual countries to be linked to public databases on the extent of COVID-19 testing in those settings [75]. The uneven global distribution of social research on testing (focused on North America and Europe) is probably a reflection of which countries have had the capacity to fund social science research on COVID-19 testing and the reduced mobility of scholars from well-funded universities during the pandemic. It may also reflect our search criteria, which excluded articles published in languages other than English. A recent bibliometric study similarly found that studies carried out in the US dominate the COVID-19 evidence base, although the study also found research from China, Italy, and India to be well represented [76]. The dominance of the evidence base by North America and Europe is especially concerning in light of vaccine inequality and at a time when the risk of future waves sweeping through the poorest populations is becoming ever more apparent. The limited scope of the existing evidence base also risks decisions being made about testing programs in some countries that fail to take account of their specific institutions, infrastructural resources, and cultural values. Especially salient in this regard is the lack of social science research in settings where testing resources are scarce but hospitalization and mortality rates are high [77].

The evidence revealed by this rapid review reflects a particular moment in the pandemic, with the second quarter of 2020 the most common period of data collection and few studies mapping changes over time. Incentives and barriers to testing are likely to change as testing technologies develop (including routine lateral flow testing becoming more widespread) and as new use cases are identified. Longitudinal research into people’s perceptions and experiences of testing at different stages of the pandemic will be crucial for establishing how generalizable the findings of this review are. It is especially important to consider the changing role of testing in a national response following the rollout of a vaccine program; and most crucially, to consider the question of whether members of the public will continue to accept the costs and burdens of testing and self-isolation once they have been vaccinated and once links between case numbers and hospitalization have been weakened, especially if structural support for these actions is not improved.

### 6.2. Limitations of the Review

This rapid review aimed to identify the scope of social science research on COVID-19 testing and to identify key common themes emerging across an interdisciplinary and rapidly changing field. It was not intended to provide a comprehensive overview of all research on this topic, nor did we undertake a systematic review of the evidence relating to specific empirical research questions (such as the impact of demographic factors on testing uptake or post-testing behaviors). Nonetheless, within the scope of its stated aims, the study has several limitations.

First, the limit of our search for certain keywords means that this review should not be taken as a comprehensive overview of all research undertaken on this topic to date. We may have missed some studies in which testing was a factor but not a core focus of the research. For example, there is a substantial and growing literature on the social factors influencing quarantine and voluntary isolation beyond their relationship to testing. Nonetheless, it is likely that our search method captured everything suitably focused on social factors related to COVID-19 testing.

Second, the inclusion of preprint articles that have not yet been approved by peer review means that some of the research reviewed may be of low quality or contain errors. The findings of this review should therefore be treated with caution. Furthermore, since the focus of the study was the scope of current research and common thematic findings rather than the evaluation of a specific evidence base, we did not undertake a quality assurance process as part of the selection protocol.

Third, for resource reasons, our search was limited to literature written in the English language. This probably excluded a large number of studies on countries outside of North America and Europe. Further research is needed to uphold our finding that the scope of research on this topic is currently largely limited to studies based in these two continents. The eligibility criteria for the review also excluded research published in report form, meaning that a substantial body of research undertaken by third-sector organizations or journalists was not represented in this study.

Fourth, and perhaps most important, is the fact that at the time of publication, this review is already likely to be superseded by both new research and new events. It is probable that knowledge, attitudes, and behaviors related to COVID-19 testing will continue to change significantly over the coming months and years. It will therefore be important to update this review regularly to establish how the scope of social science research into COVID-19 testing is changing; answer any new questions that are emerging as priorities for policy or social scientists; and explore how knowledge, attitudes, and behaviors relating to COVID-19 in different pandemic settings and health systems are changing.

## 7. Conclusions

The findings of this review suggest that testing should be understood as a social process that is inseparable from processes of contact tracing and isolation and is embedded in people’s broader experience of life during a pandemic. Given the importance of people’s motivations to seek testing of social factors—including the establishment of norms and expectations among peers, the desire to protect loved ones, and the sense of moral duty to adhere to guidelines—it is crucial that future research fully contextualizes testing in people’s social and economic circumstances and relationships. This will require in-depth qualitative research and longitudinal, observational, and/or ethnographic studies that have the capacity to identify changes in attitudes and behaviors both between moments of pre-testing, testing, and post-testing and in response to further changes in testing policies and use cases.

To further this aim, it is of paramount importance that more research is carried out among populations already identified as being most vulnerable to COVID-19—including people in lower socio-economic income groups; residents of institutions like prisons and care homes; and people from Black, Asian, and other minority ethnic backgrounds—alongside research that critically interrogates how and by whom vulnerability is defined. Furthermore, for testing to be a globally effective tool in the next stage of the pandemic, research must be rapidly undertaken to understand the barriers and incentives to testing and to adherence to post-testing guidelines in less-resourced health systems and settings with no public safety net.

There is an opportunity in many countries to build on public willingness to contribute to a communal response. However, as the pandemic continues and as socio-economic vulnerabilities and inequalities potentially intensify, there is a risk that this social contract becomes more fragile. Future COVID-19 testing policies and public health messaging must therefore actively seek to protect and nurture the willingness of members of the public to make personal sacrifices for the public good.

## Figures and Tables

**Table 1:** Characteristics of included studies.

Authors	Methods/Site/Setting	Numbers of Participants	Target Population	Type of Test	Data Collection Period	Scope	Publication Stage
**Ali et al.** [56]	Methods: Cross-sectional survey Site: Community Setting: North America	6378	National population	Unspecified	Qr2 2020	Facilitators/Barriers	Published
**Allen et al.** [31]	Methods: Longitudinal survey Site: Community Setting: North America	4759	National population; Service-users	Molecular	Qr2 2020	Testing effects	Published
**Atchison et al.** [64]	Methods: Focus groups; Interviews; Cross-sectional survey Site: Community Setting: Europe	37 in focus groups; 25 interviewed; 11711 surveyed	National population	Antibody	Qr2 2020	Facilitators/Barriers	Published
**Bender et al.** [34]	Methods: Interviews; Cohort study; Cross-sectional survey Site: Institution Setting: North America	318 surveyed; 242 interviewed	Vulnerable group (patient)	Molecular	Qr2 2020	Testing effects	Published
**Blake et al. (1)** [23]	Methods: Cross-sectional survey; Interviews; Focus groups Site: Institution Setting: Europe	99 surveyed; 41 interviewed	Student and staff group	Antibody; Molecular	Qr4 2020	Facilitators/Barriers; Testing effects	Published
**Blake et al. (2)** [27]	Methods: Focus groups Site: Institution Setting: Europe	25	Student group	Antigen	Qr4 2020	Facilitators/Barriers	Published
**Bonner et al.** [48]	Methods: Longitudinal survey Site: Community Setting: Oceania	1369	National population	Molecular	Qr2 2020; Qr3 2020	Facilitators/Barriers	Preprint
**Christensen et al.** [28]	Methods: Interviews Site: Institution Setting: Europe	15	Service-users	Molecular	Qr2 2020; Qr3 2020	Facilitators/Barriers; Testing effects	Published
**Clipman et al.** [47]	Methods: Cross-sectional survey Site: Community Setting: North America	3058	Geographic sub-population	Molecular	Qr3 2020; Qr4 2020	Facilitators/Barriers	Preprint
**Dai et al.** [59]	Methods: Cross-sectional survey Site: Community Setting: Asia	669	National population	Unspecified	Qr2 2020	Facilitators/Barriers; Testing effects	Published
**De camargo** [40]	Methods: Interviews site: Institution setting: Europe	18	Employment group	Antibody; Antigen; Molecular	Qr2 2020	Facilitators/Barriers	Published
**Earnshaw et al.** [67]	Methods: Cross-sectional survey Site: Community Setting: North America	980	National population	Unspecified	Qr2 2020	Facilitators/Barriers	Published
**Fabella** [49]	Methods: Cross-sectional survey Site: Community Setting: Asia	147	National population	Unspecified	Qr3 2020	Facilitators/Barriers	Preprint
**Ferree et al.** [54]	Methods: Case-control survey Site: Community Setting: Africa	4641	National population	Unspecified	Qr2 2020	Facilitators/Barriers	Published
**Gillam et al.** [25]	Methods: Cross-sectional survey Site: Institution Setting: Europe	458	Student group	Molecular	Unspecified	Facilitators/Barriers	Published
**Graham et al.** [30]	Methods: Case-control survey Site: Community Setting: Europe; North America	3193	Service-users; Multi-country	Molecular	Qr1 2020; Qr2 2020; Qr3 2020; Qr4 2020	Facilitators/Barriers	Preprint
**Hofschulte-Beck et al.** [37]	Methods: Cross-sectional survey Site: Institution Setting: North America	242	Healthcare workers	Molecular	Qr2 2020	Facilitators/Barriers	Published
**Kawamura et al.** [35]	Methods: Cross-sectional survey Site: Institution Setting: Asia	297	Vulnerable group (patient); Healthcare workers	Molecular	Qr2 2020; Qr3 2020	Testing effects	Published
**Kernberg et al.** [43]	Methods: Time series of cross-sectional surveys Site: Institution Setting: North America	270	Vulnerable group (patient)	Unspecified	Qr2 2020	Facilitators/Barriers	Published
**Khalifa et al.** [46]	Methods: Cross-sectional survey Site: Community Setting: Asia	664	Geographic sub-population	Unspecified	Qr4 2020	Facilitators/Barriers	Published
**Knight et al.** [41]	Methods: Interviews Site: Community Setting: North America	94	Vulnerable group	Antigen; Molecular	Qr3 2020; Qr4 2020	Facilitators/Barriers	Preprint
**Lan et al.** [36]	Methods: Cross-sectional survey Site: Institution; Community Setting: Asia	1167	National population; Healthcare workers	Molecular; Antibody; Antigen	Qr2 2020; Qr3 2020	Facilitators/Barriers; Testing effects	Published
**Lecouturier et al.** [61]	Methods: Focus groups Site: Community Setting: Europe	60	National population	Antibody	Qr2 2020	Facilitators/Barriers	Published
**Li et al.** [51]	Methods: Cross-sectional survey Site: Community Setting: North America	979	National population	Unspecified	Qr2 2020	Facilitators/Barriers; Testing effects	Published
**Ljubic et al.** [39]	Methods: Cross-sectional survey Site: Institution Setting: Europe	200	Employment group	Antibody	Qr2 2020	Testing effects	Published
**Martin et al.** [22]	Methods: Cross-sectional survey Site: Community Setting: Europe	524	Service-users	Antigen; Molecular	Qr4 2020; Qr1 2021	Testing effects	Published
**Mcelfish et al.** [50]	Methods: Cross-sectional survey Site: Community Setting: North America	1221	Geographic sub-population	Molecular	Qr3 2020	Facilitators/Barriers	Published
**Mcgowan et al.** [42]	Methods: Case-control survey Site: Community Setting: Asia	222	Vulnerable group	Unspecified	Qr3 2020; Qr4 2020	Facilitators/Barriers	Published
**Missel et al.** [33]	Methods: Interview Site: Institution Setting: Europe	15	Healthcare workers	Unspecified	Qr1 2020; Qr2 2020	Facilitators/Barriers; Testing effects	Published
**Oladoyin et al.** [53]	Methods: Cross-sectional survey Site: Community Setting: Africa	524	National population	Unspecified	Unspecified	Facilitators/Barriers	Preprint
**Ortega-Paredes et al.** [44]	Methods: Cross-sectional survey Site: Community Setting: South America	1656	National population	Antibody; Antigen; Molecular	Qr3 2020	Facilitators/Barriers	Published
**Packel et al.** [26]	Methods: Cohort study; Cross-sectional survey Site: Institution Setting: North America	2180	Student group	Molecular	Qr2 2020; Qr3 2020	Facilitators/Barriers	Published
**Ravert et al.** [32]	Methods: Cross-sectional survey Site: Community Setting: North America	178	Age-based sub- population	Unspecified	Qr3 2020	Facilitators/Barriers	Published
**Rojek et al.** [29]	Methods: Cross-sectional survey Site: Community Setting: Oceania	1846	Patient group	Molecular	Qr1 2020	Facilitators/Barriers	Published
**Siegler et al.** [63]	Methods: Cross-sectional survey Site: Community Setting: North America	1435	National population	Unspecified	Qr1 2020	Facilitators/Barriers	Published
**Smith et al.** [45]	Methods: Time series of cross-sectional surveys Site: Community Setting: Europe	53880	National population	Antigen; Molecular	Qr1 2020; Qr2 2020; Qr3 2020; Qr4 2020	Testing effects	Published
**Thunström et al.** [52]	Methods: Cross-sectional survey Site: Community Setting: North America	897	National population	Unspecified	Unspecified	Facilitators/Barriers	Published
**Trudeau et al.** [66]	Methods: Cross-sectional survey Site: Community Setting: South America	5504	Multi-country	Unspecified	Qr1 2020; Qr2 2020	Facilitators/Barriers	Published
**Valentine-Graves et al.** [65]	Methods: Cross-sectional survey Site: Community Setting: North America	148	National population	Antibody; Molecular	Unspecified	Facilitators/Barriers	Published
**Vandrevala et al.** [55]	Methods: Cross-sectional survey Site: Community Setting: Europe	778	National population	Unspecified	Qr2 2020	Facilitators/Barriers	Preprint
**Wallis et al.** [57]	Methods: Cross-sectional survey Site: Community Setting: Europe	96	Geographic sub-population	Molecular	Qr1 2020	Testing effects	Published
**Wanat et al.** [38]	Methods: Interviews; Cross-sectional survey Site: Institution Setting: Europe	18 interviewed; 214 surveyed	Students and staff group; Service-users	Antigen	Qr4 2020; Qr1 2021	Testing effects	Preprint
**Watson et al.** [24]	Methods: Interviews; Focus groups Site: Community Setting: Europe	223	Geographic sub-population; Staff and student group	Molecular (RT-LAMP)	Qr2 2020; Qr3 2020; Qr4 2020	Testing effects	Preprint
**Weiss et al.** [21]	Methods: Cross-sectional survey Site: Community Setting: North America	1421	Health service users	Molecular	Unspecified	Facilitators/Barriers	Published
**Wu et al.** [58]	Methods: Cross-sectional survey Site: Community Setting: North America	4240	National population	Molecular	Qr2 2020	Facilitators/Barriers	Published
**Zhang et al.** [60]	Methods: Case-control survey Site: Community Setting: North America	1194	National population	Unspecified	Qr3 2020	Testing effects	Preprint
**Zimba et al.** [62]	Methods: Discrete choice experiment Site: Community Setting: North America	4793	National population	Antibody; Antigen; Molecular	Qr3 2020	Facilitators/Barriers	Published

**Table 2:** Acceptability, uptake, and barriers to testing.

Themes	Findings	Studies
**Test knowledge and symptom interpretation**	General public has knowledge of main COVID-19 symptoms and/or testing types, uses, and accuracy.	Lan et al. (National pop/Healthcare workers China) Oladoyin et al. (National pop Nigeria) Ortega-Paredes et al. (National pop Ecuador) Fabella (National pop, Philippines)
	Patient interpretation of severity, type, and number of symptoms influences test-seeking behavior. Misrecognition or misattribution of symptoms to other causes is a barrier to testing.	Clipman et al. (Geographic sub pop, US) Fabella (National pop, Philippines) Graham et al. (Service-users, UK/US) Khalifa et al. (Geographic sub pop, Saudi Arabia) Mcgowan et al. (Vulnerable group, Bangladesh) Ortega-Paredes et al. (National pop Ecuador) Smith et al. (National pop, UK)
**Perceived benefits of testing**	To protect family, colleagues, and others in the community by reducing the spread of COVID-19.	Blake et al. (1) (Student and staff group, UK) Christensen et al. (Service-users, Denmark) De Camargo (Employment group, UK) Kawamura et al. (Patient group/Healthcare workers, Japan) Ljubić et al. (Employment group, Croatia) Lecouturier et al. (National pop, UK) Martin et al. (National pop, UK) Thunstrom et al. (National pop, US) Vandrevala et al. (National pop, UK) Wanat et al. (Student and staff group/service-users, UK)
	To be informed of one’s disease status.	Clipman et al. (Geographic sub pop, US) Lecouturier et al. (National pop, UK) Martin et al. (National pop, UK)
	To contribute to scientific research and public management of the pandemic.	Blake et al. (1) (Student and staff group, UK) Lecouturier et al. (National pop, UK) Watson et al. (Geographic sub-pop/Staff and student group, UK)
**Logistics of testing**	Logistical issues, including not knowing where to go to get tested, lacking transport, perceptions of long waiting times, or test turnaround times are barriers to testing.	Clipman et al. (Geographic sub pop, US) Graham et al. (Service-users, UK/US) Mcelfish et al. (Geographic sub-population, US) Zimba et al. (National pop, US)
	COVID-19 self-test kits with different sample extraction methods (cheek swab or spit, pharyngeal swab, nasopharyngeal swab, fingerprick, DBS) are deemed usable and acceptable.	Atchison et al. (National pop, UK) Gillam et al. (Student group, UK) Martin et al. (National pop, UK) Siegler et al. (National pop, US) Valentine-Graves et al. (National pop, US) Zimba et al. (National pop, US)
	People experience physical discomfort when using COVID-19 tests, in particular nasopharyngeal sampling methods.	Hofschulte-Beck et al. (Healthcare workers, US) Kawamura et al. (Patient group/Healthcare workers, Japan) Kernberg et al. (Patient group, US) Martin et al. (National pop, UK) Zimba et al. (National pop, US)
	A wide variety of test sites (school/university, workplace, drive-thru, mobile testing services, home testing) are found to be convenient. Perceived convenience of testing site encourages uptake.	Blake et al. (2) (Student group, UK) Atchison et al. (National pop, UK) Gillam et al. (Student group, UK) Knight et al. (Vulnerable group, US) Siegler et al. (National pop, US) Weiss et al. (Health service-users, US) Zimba et al. (National pop, US)
**Social pressures**	Concerns about how employers, colleagues, or peers will react, or widespread disease stigma in some settings, are barriers to testing.	De Camargo (Employment group, UK) Earnshaw et al. (National pop, US) Ferree et al. (National pop, Malawi) Khalifa et al. (Geographic sub pop, Saudi Arabia) Smith et al. (National pop, UK) Wanat et al. (Student and staff group/service-users, UK)
	Endorsement from employers, educational institutions, peers, and/or colleagues encourages testing.	Blake et al. (1) (Student and staff group, UK) Missel et al. (Healthcare workers, Denmark) Packel et al. (Student group, US) Ravert et al. (Young adults, US) Watson et al. (Geographic sub-pop/Staff and student group, UK)
**Financial burden of testing**	No association between socioeconomic variables and people’s willingness to test.	Thunstrom et al. (National pop, US) Vandrevala et al. (National pop, UK)
	Perceptions of test affordability, costs of accessing testing, and/or costs associated with self-isolation are barriers to testing.	Ali et al. (National pop, US) Graham et al. (Service-users, UK/US) Hofschulte-Beck et al. (Healthcare workers, US) Smith et al. (National pop, UK) Watson et al. (Geographic sub-pop/Staff and student group, UK)
**Trust**	Lack of trust in government bodies to deliver and manage testing is a barrier to testing in some sub-populations.	Ferree et al. (National pop, Malawi) Mcgowan et al. (Vulnerable group, Bangladesh) Watson et al. (Geographic sub-pop/Staff and student group, UK)
	The accuracy and reliability of tests are a concern for some people, and in some instances, affect willingness to test.	Knight et al. (Vulnerable group, US) Lecouturier et al. (National pop, UK)
**Vulnerable groups**	Vulnerabilities relating to health, socioeconomic status, housing, or political status can prevent people from engaging with testing services.	Bender et al. (Vulnerable patient group, US) Bonner et al. (National pop, Australia) Kawamura et al. (Vulnerable patient group/Healthcare workers, Japan) Kernberg et al. (Vulnerable patient group, US) Knight et al. (Vulnerable group, US)

**Table 3:** Impacts of testing.

Themes	Findings	Studies
**Mental health and wellbeing**	Testing is perceived as helpful for managing anxiety or fear. Receiving a negative test result is especially reassuring for individuals.	Dai et al. (National pop, Malaysia) Blake et al. (1) (Student and staff group, UK) De Camargo (Employment group, UK) Ljubić et al. (Employment group, Croatia) Kawamura et al. (Patient group/Healthcare workers, Japan) Wanat et al. (Student and staff group/service-users, UK) Watson et al. (Geographic sub-pop/Staff and student group, UK)
	Perceptions of testing as unavailable have negative effects on mental health.	Dai et al. (National pop, Malaysia)
**Adherence to guidelines**	People report high levels of adherence to guidelines on protective behaviors and/or express intentions to self-isolate should they receive a positive test result.	Christensen et al. (Service-users, Denmark) Missel et al. (Healthcare workers, Denmark) Li (National pop, US) Wallis et al. (Geographic sub-pop, UK) Wanat et al. (Student and staff group/service-users, UK) Zhang et al. (National pop, US)
	Some people find guidelines on self-isolation following a positive test result unclear or are practically unable to comply with these due to living arrangements or work.	Allen et al. (National pop/service-users, US) Blake et al. (2) (Student group, UK) Martin et al. (National pop, UK) Smith et al. (National pop, UK)
	People report that they would not change their behavior or intend to do so following a negative test (or positive in the case of an antibody test result).	Lecouturier et al. (National pop, UK) Ljubić et al. (Employment group, Croatia) Wanat et al. (Student and staff group/service-users, UK)
	Some people report engaging in riskier behaviors or intending to do so following a negative test result.	Martin et al. (National pop, UK) Zhang et al. (National pop, US)

## Supplementary Materials

The following are available online at https://www.mdpi.com/article/10.3390/diagnostics11091685/s1, An overview of search strings, dates and inclusion criteria, including Table S1., Databases and search strategy, and Table S2., Inclusion and exclusion criteria.
