# Supplementary material for: Knowledge, Attitudes, and Behavior Related to COVID-19 Testing: A Rapid Scoping Review
Source: Diagnostics (Basel). 2021 Sep 15;11(9):1685. doi: 10.3390/diagnostics11091685 (PMC8472251; doi:10.3390/diagnostics11091685)
Supplement: Supplementary file 1 [file diagnostics-11-01685-s001.zip › diagnostics-1331847-supplementary.pdf]

Supplementary Materials

Search strings and dates

Basic searches were conducted unless otherwise stated, then an advance search of the topic was used. Databases were searched for results for the period 2020—26 March 2021. The search was enhanced by running forward citation checks and consulting the bibliographic references of relevant articles. The following databases were used:

Table S1. Databases and search strategy.

	Resources Searched	Keywords/ Search Strategy (Combined with AND)
Jan 2020–26 May 2021	Web of Science Core Collection Medline Scopus	TITLE = (covid* OR corona* OR SARS*) TITLE = (test* OR screen* OR diagnos*) TITLE-ABS-KEY = (qualitative OR interview* OR survey*) TITLE-ABS-KEY = (expectation* OR trust* OR experience* OR attitude* OR barrier* OR perception* OR behaviour* OR behavior* OR view* OR adher* OR perceive* OR compliance OR comply* OR understand* OR enable* OR intention* OR belief* OR believe*)
	WHO Covid-19 Global Literature	(ti:(covid OR coronavirus OR corona OR sars)) AND (ti:(test OR tests OR testing OR tested OR screen OR screening OR diagnostic OR diagnose OR diagnosis)) AND (tw:(qualitative OR interview OR interviewed OR interviews OR surveyed OR survey OR surveys)) AND (tw:(expectations OR trust OR experiences OR attitudes OR barriers OR perception OR perceptions OR perceived OR perceive OR behaviour OR behaviours OR behavior OR behaviors OR view OR views OR adhere OR perceive OR compliance OR comply OR understand OR understanding OR enable OR enabler OR intention OR intentions OR belief OR believe OR adherence))
**26 May–31 May 2021**	**Google Scholar**	Used for forward citation checks only

Web of science core collection searched 26 March 2021
= 92 results

TI = (covid* OR corona* OR SARS*)

TI = (test* OR screen* OR diagnos*)

TS = (qualitative OR interview* OR survey*)

TS = (expectation* OR trust* OR experience* OR attitude* OR barrier* OR perception* OR behaviour* OR behavior* OR view* OR adher* OR perceive* OR compliance OR comply* OR understand* OR enable* OR intention* OR belief* OR believe*)

Scopus searched 26 March 2021
=155 results

(TITLE (test* OR screen* OR diagnos*) AND PUBYEAR > 2019) AND (TITLE (covid* OR corona* OR sars*) AND PUBYEAR > 2019) AND (TITLE-ABS-KEY (qualitative OR interview* OR survey*) AND PUBYEAR > 2019) AND (TITLE-ABS-KEY (expectation* OR trust* OR experience* OR attitude* OR barrier* OR perception* OR behaviour* OR behavior* OR view* OR adher* OR perceive* OR compliance OR comply* OR understand* OR enable* OR intention* OR belief* OR believe*) AND PUBYEAR > 2019)

Medline searched 26 March 2021
= 77 results

TI = (covid* OR corona* OR SARS*)

TI = (test* OR screen* OR diagnos*)

TS = (qualitative OR interview* OR survey*)

TS = (expectation* OR trust* OR experience* OR attitude* OR barrier* OR perception* OR behaviour* OR behavior* OR view* OR adhere* OR perceive* OR compliance OR comply* OR understand* OR enable* OR intention* OR belief* OR believe*)

WHO Covid-19 Global Literature searched 26 March 2021
=150 results

(ti:(covid OR coronavirus OR corona OR sars)) AND (ti:(test OR tests OR testing OR tested OR screen OR screening OR diagnostic OR diagnose OR diagnosis)) AND (tw:(qualitative OR interview OR interviewed OR interviews OR surveyed OR survey OR surveys)) AND (tw:(expectations OR trust OR experiences OR attitudes OR barriers OR perception OR perceptions OR perceived OR perceive OR behaviour OR behaviours OR behavior OR behaviors OR view OR views OR adhere OR perceive OR compliance OR comply OR understand OR understanding OR enable OR enabler OR intention OR intentions OR belief OR believe OR adherence))

Notes on Process

- An initial general round of basic searches was run (e.g., covid AND test). Results were unwieldy (>1000).
- A second round of searches was run with:

TITLE-ABS-KEY = (covid* OR corona* OR SARS*)

TITLE-ABS-KEY = (test* OR screen* OR diagnos*)

TITLE-ABS-KEY = (qualitative OR interview* OR survey*)

TITLE-ABS-KEY = (expectation* OR trust* OR experience* OR attitude* OR barrier* OR perception* OR behaviour* OR behavior* OR view* OR adher* OR perceive* OR compliance OR comply* OR understand* OR enable* OR intention* OR belief* OR believe*)

This generated a substantial number of papers (between 300-888 depending on the database) that did not meet our criteria for relevance.

- To ensure we were finding the most relevant papers for purposes of rapid review, we changed the two first parts of our search string to limit our findings to papers containing references to both covid and testing in their titles. A third round of searches was run with:

TITLE = (covid* OR corona* OR SARS*)

TITLE = (test* OR screen* OR diagnose*)

TITLE-ABS-KEY = (qualitative OR interview* OR survey*)

TITLE-ABS-KEY = (expectation* OR trust* OR experience* OR attitude* OR barrier* OR perception* OR behaviour* OR behavior* OR view* OR adhere* OR perceive* OR compliance OR comply* OR understand* OR enable* OR intention* OR belief* OR believe*)

This generated 77-155 papers (depending on the database) which better aligned with our criteria for relevance.

- The databases returned 474 papers, which were uploaded to Covidence. Two hundred sixty-three duplicates were removed in total, 48 of which were identified and removed manually by the reviewers following an initial removal of duplicates by the software. The titles and abstracts of 211 articles were screened for relevance using inclusion/exclusion criteria (Table 5) by two independent reviewers. Any conflicts between reviewer one and reviewer two were resolved.
- 65 full texts were reviewed by the same two reviewers using the same criteria.
- 38 articles met the inclusion criteria.

The following inclusion and exclusion criteria were applied:

Table S2. Inclusion and exclusion criteria.

	**Inclusion Criteria**	**Exclusion Criteria**
Study design	Primary empirical research (qualitative or quantitative), evaluation or secondary reviews	Primarily theoretical or conceptual in nature, lacking empirical evidence or explanation of methodology
Language	English	Studies published in another language than English
Publication date	From 2020	Prior to 2020

Publication format	Journal articles, peer-reviewed materials, articles under review, published books and book chapters, other academic research, research commissioned by governments	Opinion or statement pieces, magazine articles, blog posts, unpublished reports.
Study aim	Studies exploring attitudes, expectations, experiences of and trust in testing for people targeted for COVID-19 testing	Studies focusing on general prevalence, testing for other diseases, providers of testing

- 9 additional articles were identified by running forward citation checks and in-text citation checks between 26 March and 31 March 2021 and were screened for eligibility by two reviewers.

The scoping review analyzed these 47 papers.
